# Recent advances in exosome-mediated nucleic acid delivery for cancer therapy

**DOI:** 10.1186/s12951-022-01472-z

**Published:** 2022-06-14

**Authors:** Ying Zhang, Qiqi Liu, Xinmeng Zhang, Haoqiang Huang, Shiqi Tang, Yujuan Chai, Zhourui Xu, Meirong Li, Xin Chen, Jia Liu, Chengbin Yang

**Affiliations:** 1 Central Laboratory of Longgang District People’s Hospital of Shenzhen & The Second Affiliated Hospital, The Chinese University of Hong Kong, Shenzhen, 518172 China; 2 Guangdong Key Laboratory for Biomedical Measurements and Ultrasound Imaging, School of Biomedical Engineering, Health Science Center, Shenzhen University, Shenzhen, 518060 China

**Keywords:** Gene therapy, Exosome, Nucleic acid drug, Delivery, Cancer treatment

## Abstract

Cancer is a leading public health problem worldwide. Its treatment remains a daunting challenge, although significant progress has been made in existing treatments in recent years. A large concern is the poor therapeutic effect due to lack of specificity and low bioavailability. Gene therapy has recently emerged as a powerful tool for cancer therapy. However, delivery methods limit its therapeutic effects. Exosomes, a subset of extracellular vesicles secreted by most cells, have the characteristics of good biocompatibility, low toxicity and immunogenicity, and great designability. In the past decades, as therapeutic carriers and diagnostic markers, they have caught extensive attention. This review introduced the characteristics of exosomes, and focused on their applications as delivery carriers in DNA, messenger RNA (mRNA), microRNA (miRNA), small interfering RNA (siRNA), circular RNA (circRNA) and other nucleic acids. Meanwhile, their application in cancer therapy and exosome-based clinical trials were presented and discussed. Through systematic summarization and analysis, the recent advances and current challenges of exosome-mediated nucleic acid delivery for cancer therapy are introduced, which will provide a theoretical basis for the development of nucleic acid drugs.

**Graphical Abstract:**

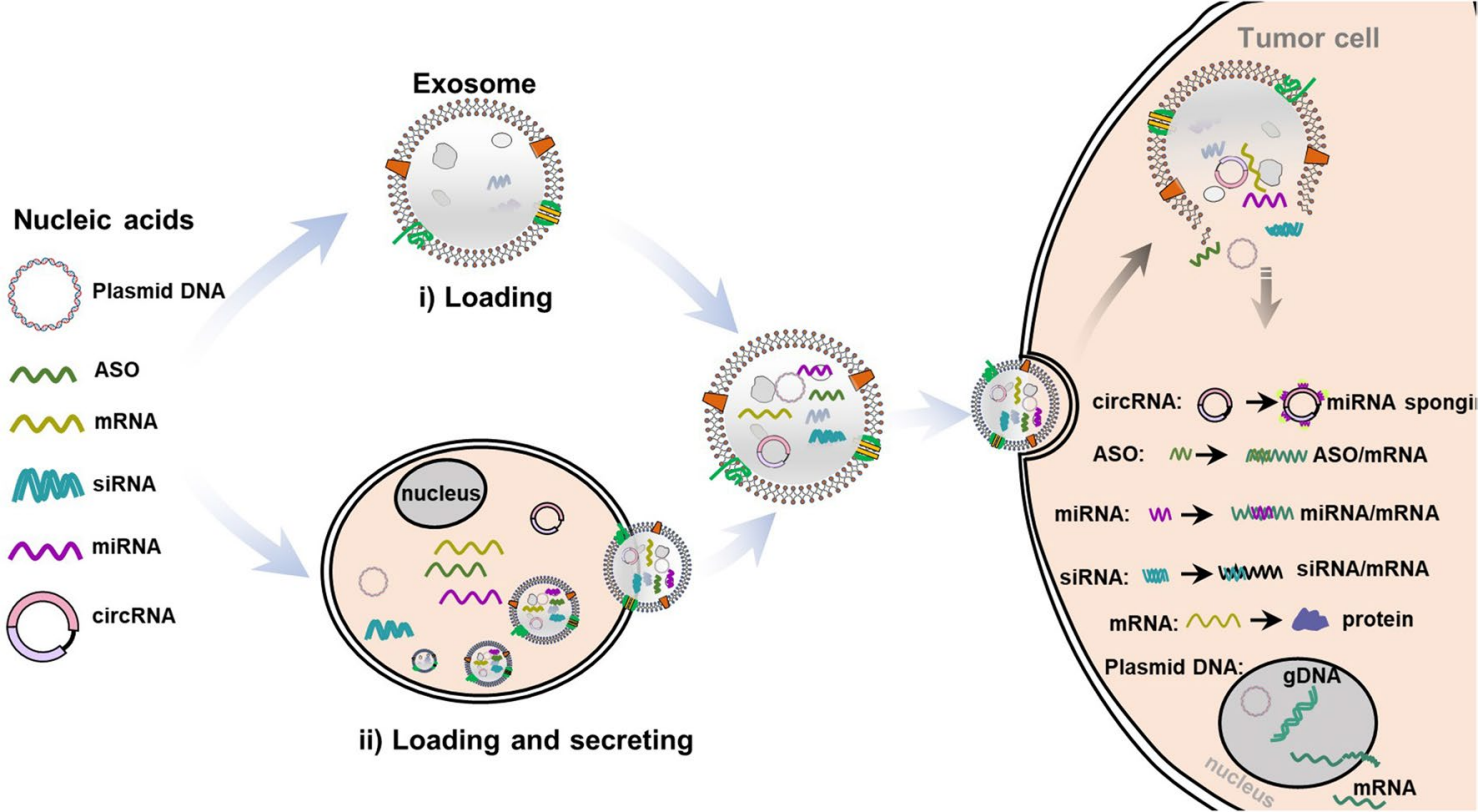

## Introduction

Cancer remains one of the leading causes of death globally, with the prevalence of 410 million mortalities annually [1]. In 2021, there had been 19.29 million patients diagnosed with cancer, and nearly 10 million people died of cancer [2]. To fight cancer, various treatments such as surgical therapy, chemotherapy, and radiotherapy have been developed. These strategies have become more focused and personalized based on the type and stage of the disease, which has led to a decline in cancer-related mortality over the past decades [3]. Despite their undisputed contribution, these invasive and/or often lacking cancer cell-selective techniques lead to a wide range of harmful side-effects, such as high recurrence rates, enormous trauma, poor survival and impaired life quality, which often hamper therapy success. Consequently, there is an urgent demand to develop safe and efficacious therapeutic techniques for treating cancer.

Gene therapy is the therapeutic delivery of genetic material into cells to compensate for abnormal genes by either turning off genes that produce faulty proteins or introducing genes to make a beneficial protein to treat disease [4–6]. It is a safe and effictive method for treating a wide range of diseases, especially for cancer. The effect of gene therapy depends on the targeting of nucleic acids drugs , the delivery efficiency, and accuracy of delivery tools. The nucleic acids including specific DNA, messenger RNA (mRNA), microRNA (miRNA), small interfering RNA (siRNA), circular RNA (circRNA), which have been widely exploited for gene therapy. However, nucleic acids are negatively charged and hydrophilic, which cannot directly penetrate cell membranes and are vulnerable to enzymatic degradation, so they cannot be effectively transported to cells [4]. In this situation, delivery systems are necessary, which cannot only prevent the nucleic acids degrading in the bloodstream and being filtered out by the kidney, but can deliver them to desired locations.

Extracellular vesicles (EVs) are small membranous vesicles released from different cells to the extracellular matrix, which can participate intercellular communication between cells [7]. According to EVs’ size and origin, they are divided into three subgroups: (1) apoptotic bodies (500 nm–5 μm) released during programmed cell death, (2) microvesicles (150–500 nm) from the budding of the plasma membrane, and (3) exosomes (40–150 nm) from endosomes [8]. Owing to their nano size, exosomes are considered as the most promising drug delivery tools. Compared to conventional delivery systems such as lipid nanoparticles (LNPs), exosomes have the following advantages: (1) Exosomes are more stable in body fluid than LNPs, because LNPs can be easily removed by macrophages or reticuloendothelial cells [9]. (2) Due to their endogenous source and high biocompatibility, exosomes have relatively low cytotoxicity and immunogenicity [10]. (3) Exosomes can provide better drug protection during delivery, because drugs are within the double-layer exosomal membrane, while drugs appeared outside the LNPs, which are easier to degrade [11]. (4) Exosomes can deliver both hydrophobic and hydrophilic molecules. And they have effective homing ability to tumor sites, which may be attributed to their multivalent display of cell-derived surface moieties [10]. (5) Exosomes derived from tumor can escape the phagocytosis of mononuclear phagocyte system through the binding of CD47 on exosomal surface and signal regulatory protein alpha (SIRPα) on the face of macrophages and sending out “don’t eat me” signal [12]. (6) Exosome can cross the blood–brain barrier and reach the brain tissue owing to their small size and characteristics [13]. (7) Exosomes have high cellular uptake and are easily modified according to the target cells owing to membrane proteins such as tetraspanin and fibronectin [10].

Herein, we summarized exosomes’ characteristics and applications as various nucleic acid (DNA, mRNA, miRNA, siRNA and circRNA) delivery carriers for cancer therapy. Meanwhile, the challenges and the prospective in using exosome-mediated nucleic acids are also discussed.

## The biogenesis of exosome

**Fig. 1:**
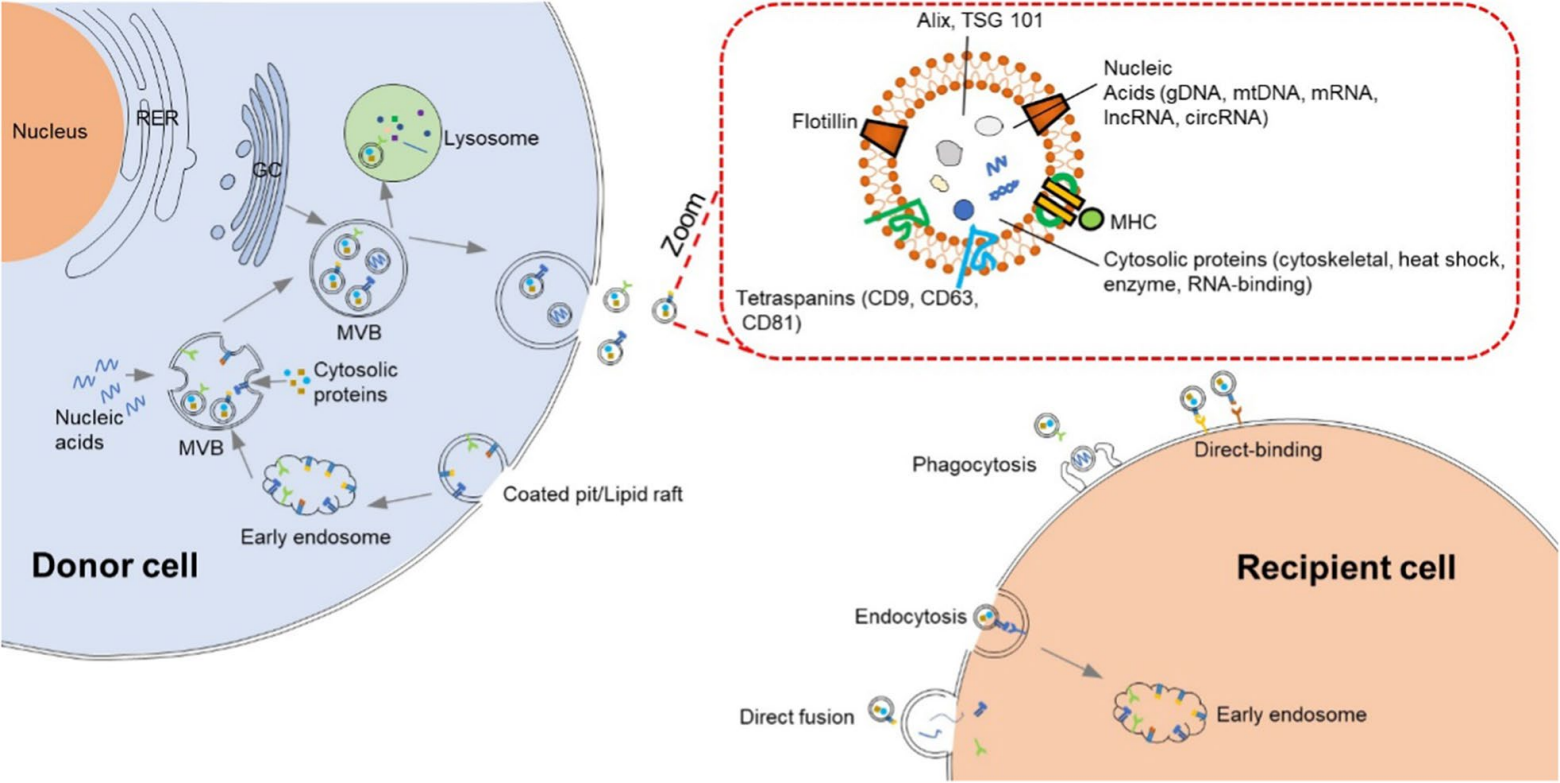
The biogenesis, contents, and internalization of exosomes

Exosomes are native nanovesicles with a diameter of 30–120 nm secreted from various cell types, including cancer cells, dendritic cells, B cells, T cells, mast cells and epithelial cells, and exist in different body fluids such as blood, urine, malignant effusions, bronchoalveolar lavage fluid and breast milk, etc. [14–17]. They were first found in the supernatant of sheep erythrocytes cultured in vitro in 1983 [18, 19]. At that time, exosomes were considered as the “Garbage Bags” for cells to eliminate unwanted products out of the cells. Subsequently, people found that they were formed by plasma membrane invagination, followed by acidification and maturation of mass exchange into the late endosomes. Late endosomes eventually form multi-vesicles, the membrane of which is sunken inward and sprouts to form intraluminal vesicles, which are exosomes. Finally, they are secreted out of the cell by fusing the plasma membrane (Fig. 1) [17]. The natural internal cargo of exosomes includes specific mRNAs, miRNAs, proteins, etc. (Fig. 1). Several proteins such as tetraspanins (CD9, CD63, CD81), heat shock proteins, and fusion proteins (flotillin) are identified on the surface of exosomes (Fig. 1). These tetraspanins could be used as a specific marker to isolate exosomes. Some studies revealed that exosomes’ target-homing capabilities depended on the surface proteins binding to receptor molecules on the target cell [20, 21]. In addition, many tumor cells secreted exosomes tenfold more than normal cells [21]. Furthermore, the presence of specific genetic information within exosomes derived from tumor cells offers opportunities to develop simple liquid biopsy-based approaches for cancer diagnosis or to monitor the effectiveness of cancer treatment [22].

## The approaches and advances in exosome-mediated delivery

The effect of exosome-mediated therapy mainly depends on the source of exosomes, the loading methods of therapeutic molecules, the efficiency of cell uptake of exosomes. Exosomes derived from different cell types have diverse functions. For example, human embryonic kidney (HEK293) cells have been widely used in the field of biopharmaceutical manufacturing owing to the advantages of easy to growth, non-needing harsh culture conditions, and high transfection efficiency [23]. Moreover, HEK293 cells can accept various transfection methods and allow gene manipulation to modify the exosomal surface or load cargos during exosomal biogenesis [24]. And exosomes derived from HEK293 are immune inert and do not trigger inflammatory reactions in vivo [23]. In addition, cancer cells can secrete a large number of exosomes, because the overexpressed Rab27a and Rab27b proteins in cancer cells are involved in the process of exosome release [25]. Cancer cell-derived exosomes have a tropism toward cell origin due to their abundant biological components similar to their parent tumor cells, which can be used for cancer targeting [26]. Qiao et al. [26] isolated exosomes from two cancer cell lines (HT1080, human fibrosarcoma cells, and Hela, human cervical cancer cells) and observed that the uptake of HT1080 exosomes in HT1080 cells was twofold that of Hela exosomes. Furthermore, in vivo therapeutic experiments revealed that the inhibition rate of HT1080 exosomes loaded with a common chemotherapy drug Doxil was threefold higher than that of Hela exosomes with Doxil. However, there are some limitations, such as an unsatisfactory pharmacokinetic profile, being involved in tumor development and metastasis, and potential safety issues, which are expected to be improved to be better used in cancer treatment [10]. Besides, exosomes derived from immune cells have also been widely studied. For instance, monocytes- and macrophages-derived exosomes have been shown to evade immune phagocytosis [27]. Dendritic cell (DC)derived exosomes hold a significant advantage as they have been proven secure in different types of cancer [28]. And these exosomes loaded with tumor antigens have been effective against non-small cell lung cancer (NSCLC) [29]. As is shown in Table 1, exosomes from different sources have different advantages and disadvantages. Therefore, the purpose of good therapy can be achieved by selecting appropriate exosomes according to therapeutic requirements.

**Table 1:** The advantages and disadvantages of different types of exosomes

Types	Sources	Advantages	Disadvantages	References
Cell-secreted exosomes	Human embryonic kidney cells	Ease of growth; non-demanding maintenance conditions; high transfection efficiency; ideal host cells for membrane modification through gene manipulation	Immune inert	[23]
	Cancer cells	Large secretion; targeting homotypic tumor	Have a less ideal pharmacokinetic profile; be involved in tumour development and metastasis; having potential safety issues	[30]
	Immune cells (e. g. macrophage cells, dendritic cells, natural killer cells)	Reduced immunogenicity; inducing potent cellular immune responses; containing killer proteins and cytotoxic molecules to inhibit tumour growth; penetrating the blood–brain barrier	Lack of understanding of mechanisms regarding how exosomal components interact with acceptor cells	[10]
	Stem cells (e. g. mesenchymal stem cells)	Immune regulation characteristics; low production cost; good homing and penetrating ability	The unclear cargo composition of exosomes and biological behavior mechanism	[31, 32]
Blood-derived exosomes	Blood	Wide source and easy access; reduced unexpected mutations in cell culture; no occurring horizontal gene transfer; high transfection efficiency; natural brain targeting ability	Not determined	[33]
Food-derived exosomes	Milk-derived exosomes	Rich sources; crossing through the gastrointestinal tract via the neonatal Fc receptor; improving the oral bioavailability of drugs; improving the effectiveness and stability of drugs; improving human and mouse intestinal cells	Variation in shape, size, and cargo contents of exosomes; the unclear mechanism of the absorption, movement, and action	[34–36]
	Plants-derived exosomes (e. g. grape, strawberry, lemon)	Rich sources; have the stability in the digestive environment	Less understanding of the ability in the process; the unclear mechanism of the absorption, movement, and action	[36, 37]

Co-incubation, transfection, and electroporation are the frequently-used methods of therapeutic molecules loading into exosomes [38]. The aqueous core and bilayer lipid membrane of the exosomes make the loading of hydrophilic and hydrophobic drugs easier through co-incubation [39]. When hydrophilic molecules fail to spontaneously pass through the lipid bilayer, loading can be achieved by liposome transfection and electroporation to form transient pores on the exosomal membrane. Transfection-based approaches have been proved to have better loading efficiency and protein stability, but they are undesirable because of their toxicity and side effects of transfectants in altering cell gene expression [40]. Electroporation has been widely used as a safer method in therapeutic molecules loading into exosomes. Shtam et al. [40] provided sufficient evidence that the nucleic acids were more effectively introduced into exosomes from HeLa cells using electroporation than chemical treatment. However, not all cell-derived exosomes can be loaded by electroporation. For example, Ohno et al. [41] found that when using HEK293T cells as the source of exosomes, liposome transfection could load nucleic acids successfully while electroporation did not. Therefore, the method may need to be optimized for each exosome and cell type. In addition, in recent years, several new loading methods are emerging gradually. For instance, an active delivery modality exploits the HIV-1 TAR and RNA-TAT peptide interaction by swapping the wild type pre-miR loop with the TAR RNA loop. The modified pre-miR is designed to recognize the TAT peptide introduced into the exosomes using a Lamp2a fusion protein. The loading of the miRNA into exosomes was enhanced using this TAT-TAR interaction [42, 43]. The modified calcium chloride (CaCl_2_) method (CaCl_2_-heat shock) has successfully loaded nucleic acids into exosomes through forming CaCl_2_-nucleic acid complex, which was absorbed by exosomes under heat shock at 42 °C [44–46]. In addition, plasmid-mediated therapeutic molecule transfer has been gradually applied. A constructed plasmid containing therapeutic molecule genes is transfected into exosome-producing cells. After culture, the exosomes produced by donor cells contain therapeutic molecules [47, 48]. Based on the above, the correct selection of therapeutic molecules loading into the exosomes can achieve unexpected therapeutic effects.

**Fig. 2:**
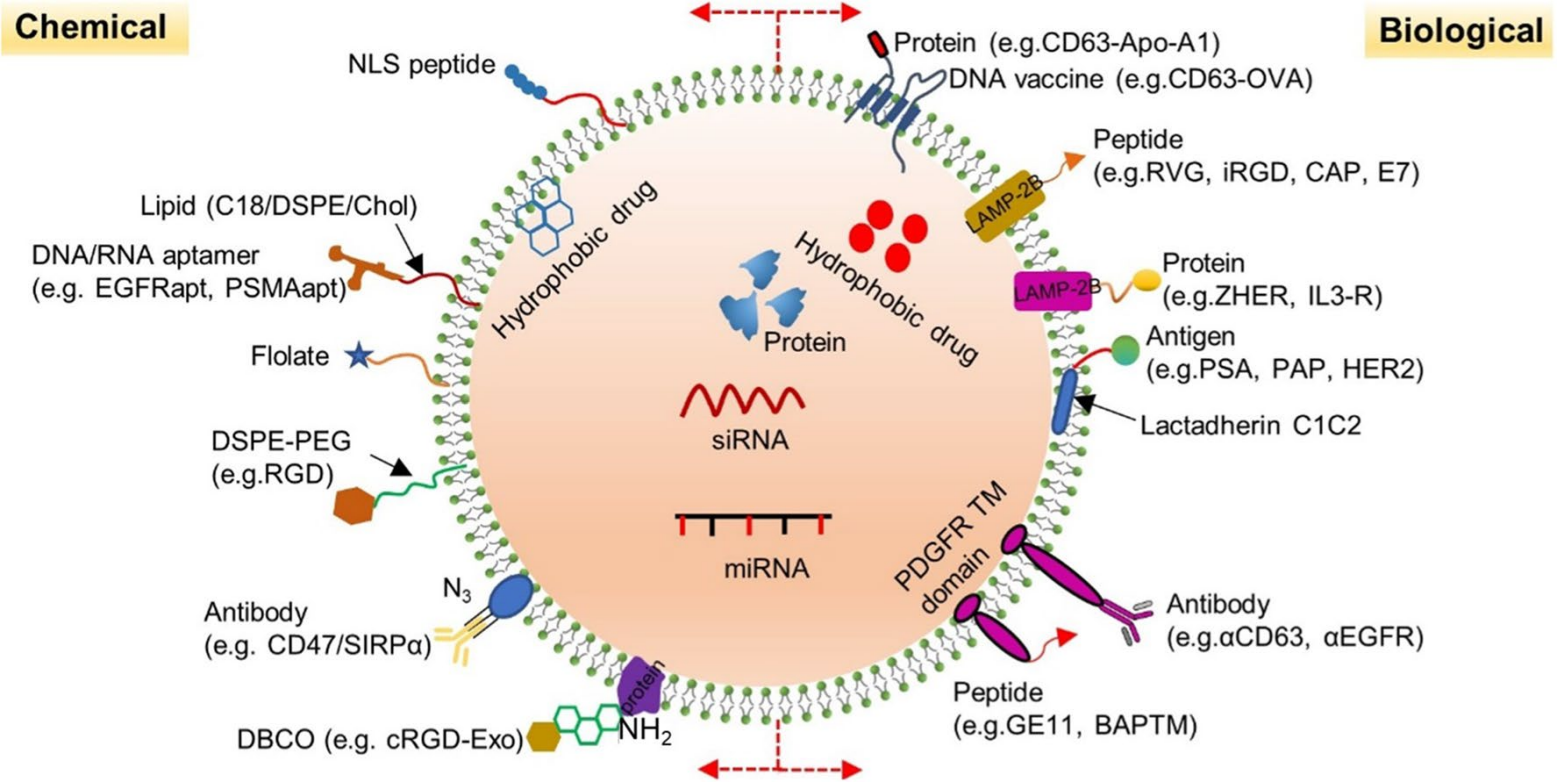
The chemical and biological modification on exosome surfaces

In addtion, exosomes loaded therapeutic molecules also face several challenges, including competition from endogenous exosomes, the internalization/clearance by the mononuclear phagocyte system, and targeting [49–51]. To solve these problems, it is essential for specific modification on exosome surfaces. These modification include chemical and biological modifications (Fig. 2). The former depends on the biological binding of targeted ligands to surface proteins, but surface protein inactivation or exosome aggregation may occur. The latter is an important strategy to display functional ligands on the exosome membrane, but it requires plasmid construction and overexpression of proteins in donor cells. Despite the defects, both methods have been successfully applied. For instance, Zhan et al. [52] constructed the the amphiphilic phosphatidylcholine (PC) exosome through inserting PC into the membrane lipid layer of the reticulocyte exosome from the blood. Compared with natural exosome, PC exosome increased the efficiency of tumor cell internalization by nearly twice. After loading therapeutic drugs, PC exosome significantly promoted the accumulation of drugs in tumor cells and showed enhanced antitumor activity in vitro. In addition, the surface of bovine serum‐derived exosomes is modified with α‐d‐mannose to facilitate interaction with mannose receptors on DCs and efficient delivery of immune stimulators to the DCs [53]. Zuo et al.[54]. added a potent adjuvant, high mobility group nucleosome-binding protein 1 (HMGN1) to tumor cell-derived exosomes, which enhanced the ability of DC to activate T cells and sustained protective immune response for about 9 weeks.

Above all, to improve the efficiency and accuracy of drug delivery to achieve good therapeutic effect, the selection of exosomes, the loading mode of therapeutic molecules and surface engineering modification of exosomes are the main factors that should be considered comprehensively. Because exosomes from different cells have different functions, choosing the right exosomes as drug delivery tools can greatly improve the targeting of drug delivery. The loading capacity of therapeutic molecules can be significantly improved by appropriate loading method. Further, the engineering modification of exosome surface can achieve the loading efficiency and targeting of therapeutic molecules at the same time. Thus, integrating various advantages to deliver drug molecules through exosome can achieve the ideal therapeutic effect.

## Exosomes-based nucleic acid delivery system for cancer treatment

### Exosomes-based DNA delivery system

#### ASOs

In addition to the well-known genomic DNA, mitochondrial DNA and plasmid DNA, antisense oligonucleotides (ASOs) are another important DNA species which are single-stranded DNA molecules and usually consist of 12–25 nucleotides, can complementarity to target mRNA [55]. Following binding to the targeted RNA, the ASOs can regulate RNA function through several mechanisms. One is that ASOs can form RNA–DNA hybrid and serve as the substrate of RNase H-mediated cleavage, leading to the hydrolysis of a hybridized RNA strand [56, 57]. The formation of ASO-RNA heteroduplex also leads to splicing inhibition or exon skipping events by spatially blocking standard splicing sites [56]. Another is that ASO only plays a space-occupying role and does not directly degrade target RNA. For instance, ASOs can be designed to bind the miRNAs and block the targeted RNA, resulting in inhibition of translation of the RNA and increase the expression of a variety of proteins [57]. In addition, ASOs are designed to bind to a regulatory sequence in the 5-untranslated region of an mRNA that represses protein translation, such as an upstream open reading frame or stem-loop structure [57]. As a powerful molecular tool, ASOs are widely used in protein and RNA biology and are a highly selective therapeutic strategy for many diseases related to gene expression disorders. So far, over ten ASOs have been approved by Food and Drug Administration (FDA) [58]. Therefore, the successful loading of specific ASOs into cells play a key role in cancer therapy.

#### Loading methods for DNA into exosomes

As a common strategy, electroporation also allows DNA to be loaded into exosomes through creating pores on exosomal lipid bilayers. It has been shown that ASO4, ASO-210 or scramble ASO loaded into exosomes by electroporation could be delivered to recipient cells and knock down specific gene expression [59–61]. However, a major disadvantage of electroporation is the formation of nucleic acid and exosome aggregates during encapsulation, which will affect the function of nucleic acids [62]. Lamichhane et al. [59] reported that exosomes carrying plasmid DNA by electroporation delivered DNA to recipient cells; however, these DNAs were not functionally active. To solve these problems, it may be necessary to optimize electroporation parameters. In addition, some new loading methods have been developed. An exosomal liposome hybrid was formed through fusing the lipid bilayer of the exosomal membrane with liposomes, which could encapsulate and deliver large DNA molecules, such as CRISPR/Cas9 plasmid, and reduce the toxicity of liposomes [63]. Exosome-associated adeno-associated virus (exo-AAV) has also been proved to be a powerful system for DNA delivery. György et al. [64] cloned a mouse-codon-optimized gene encoding lipoma HMGIC fusion partner-like 5 (LHFPL5) with a hemagglutinin (HA) tag at the N terminus into an AAV vector, and then was transfected into HEK293T cells using the calcium phosphate and obtained exo-AAV1-HA-Lhfpl5, which could rescue hearing in a mouse model of hereditary deafness. Therefore, the successful loading will provide a basis for exosome-mediated DNA delivery for cancer treatment.

#### Delivery of therapeutic DNA

**Fig. 3:**
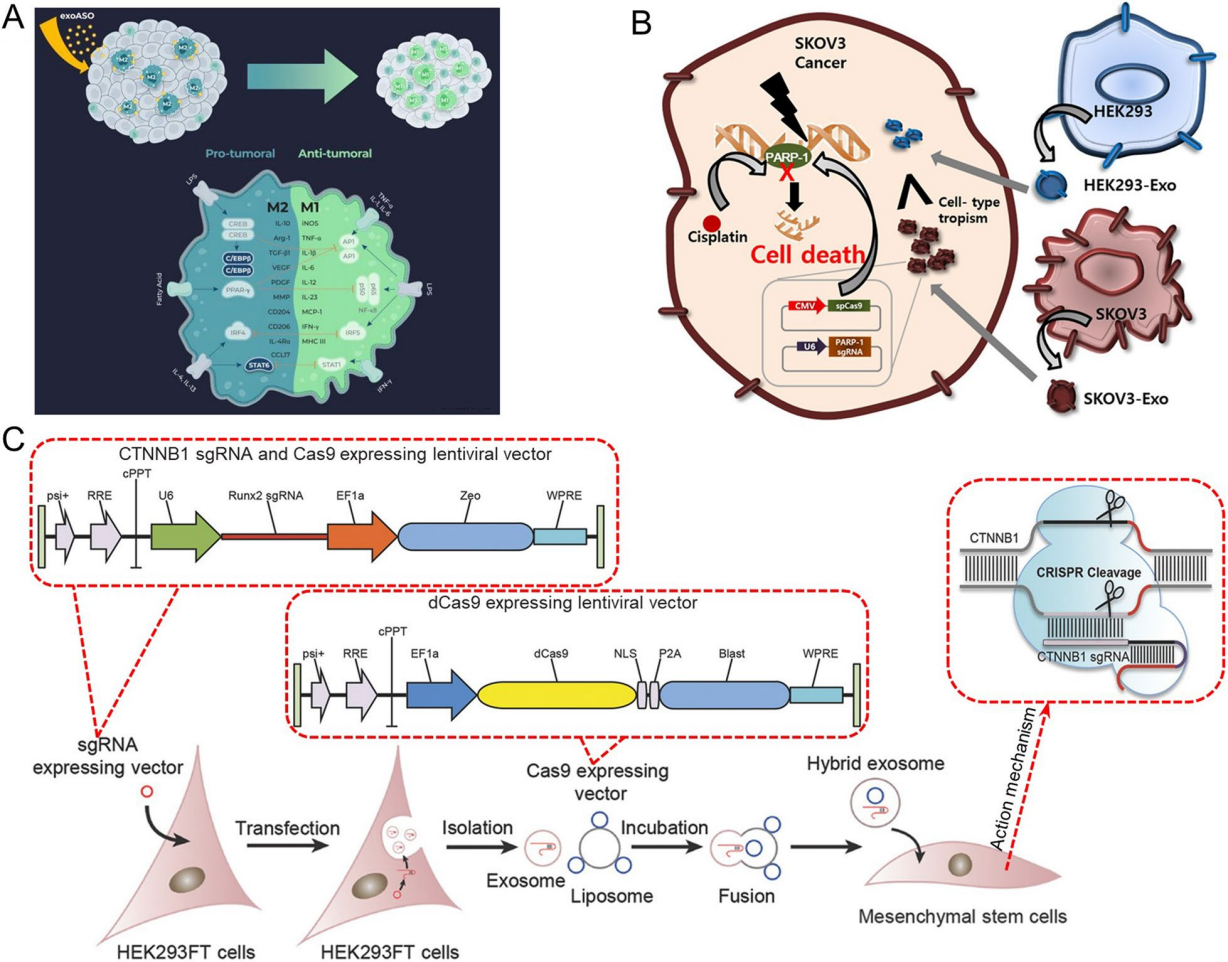
**A** Engineered exosomes to deliver ASO to produce effective antitumor activity [70]. **B** Exosomes loaded with CRISPR/Cas9 targeting PARP-1 for cancer therapy [71]. **C** The hybrid exosomes successfully deliver CRISPR/dCas9 interference system [63]

In recent years, it has been reported that exosome deliver various functionlized DNA into cells via the process of exosome-endocytosis to treat cancer [65–69]. However, due to their small size, the efficiency of packaging large DNA through exosomes is very low, which limit the application of the exosome-based drug delivery system. The relatively small ASO and plasmid DNA, or engineering modified exosomes are used to solve the problem. Codiak Biosciences [70] published the first preclinical data of engineered exosomes to deliver ASO (exoASO), demonstrating the potential of exoASO to M2 macrophages to target the expression of key immunosuppressive transcription factors STAT6 and C/EBP (Fig. 3A). The results revealed that the expression of *TNF* and *IL-10* related to exoASO therapy increased up to 40-fold and 29-fold respectively, which was consistent with the repolarization from immunosuppressive M2 macrophages to immunostimulatory M1 macrophages, and exoASO-STAT6 significantly slowed tumor growth, and tumors in 50% of mice completely subsided. When exoASO-STAT6 was combined with anti-PD1 antibody, the tumor remission rate was further improved by 25%. It was exciting that FDA has recently approved the investigational new drug application of exoASO-STAT6. In addition, with the rise of CRISPR/Cas9-mediated genome editing, the delivery of Cas9-encoding plasmid through exosome has also been tried. For example, Kim et al. [71] reported that ovarian cancer-derived exosomes (SKOV3-Exo) could be efficiently electroporated with CRISPR/Cas9 plasmids in vivo to suppress the expression of poly (ADP-ribose) polymerase-1 (PARP-1). The results suggested that compared with SKOV3-Exo alone, the expression of PARP-1 was completely inhibited after treatment with CRISPR/Cas9-loaded SKOV3-Exo, and the tumor volume in the treatment group hardly changed within 20 days of intratumoral injection treatment, while that in the control group kept growing (Fig. 3B). Besides, Lin et al. [63] developed a kind of hybrid exosomes with liposomes to deliver the CRISPR/Cas9 expression plasmids into mesenchymal stem cell (MSC) target cells, and the results revealed that hybrid nanoparticles carried the large CRISPR/Cas9 expression plasmids could down-regulate the expression of gene *Runx2* by twofold compared with the control group (only Runx2 guided CRISPR/dCas9 system) (Fig. 3C). Therefore, exosome mediated ASO or CRISPR/Cas9 plasmids into cells could correct or destroy oncogenes through regulating mRNA translation or therapeutic genome editing (gene destruction, gene correction, gene deletion, gene insert, etc.), respectively [72, 73].

### Exosomes-based mRNA delivery system

#### mRNA

mRNA is an intermediate molecule that transmits the genetic code from DNA to the ribosome for protein expression. It has been considered as another promising tool for the treatment of a variety of diseases, especially cancer [74, 75]. Compared with DNA-based therapy, RNA-based therapy is more advantageous: (a) DNA transcription must precede translation and need to enter the nucleus. The efficiency is limited because less than 0.10% of cytoplasmic DNA enters the nucleus; In contrast, mRNA is directly translated when entering the cytosol, resulting in effective gene expression [76]; (b) mRNA has no risk of genome integration and will not cause insertion mutation [77]; (c) unlike DNA, mRNA is also translated in tumor dormant cells [78]. Based on the above advantages, the use of mRNA technology to develop vaccines for related diseases, including cancer, has gradually attracted extensive attention [79]. In particular, since the outbreak of COVID-19, a variety of mRNA vaccines have been rapidly developed using mRNA technology [80–82]. Nevertheless, mRNA is easy to be degraded by nuclease, easy to activate immune response, and large (10^4^–10^6^ Da) [83], which has become the main obstacle to the development of mRNA drugs. Exosomes, as a natural delivery carrier, can realize the effective delivery of mRNA.

#### Loading methods for mRNA into exosomes

**Fig. 4:**
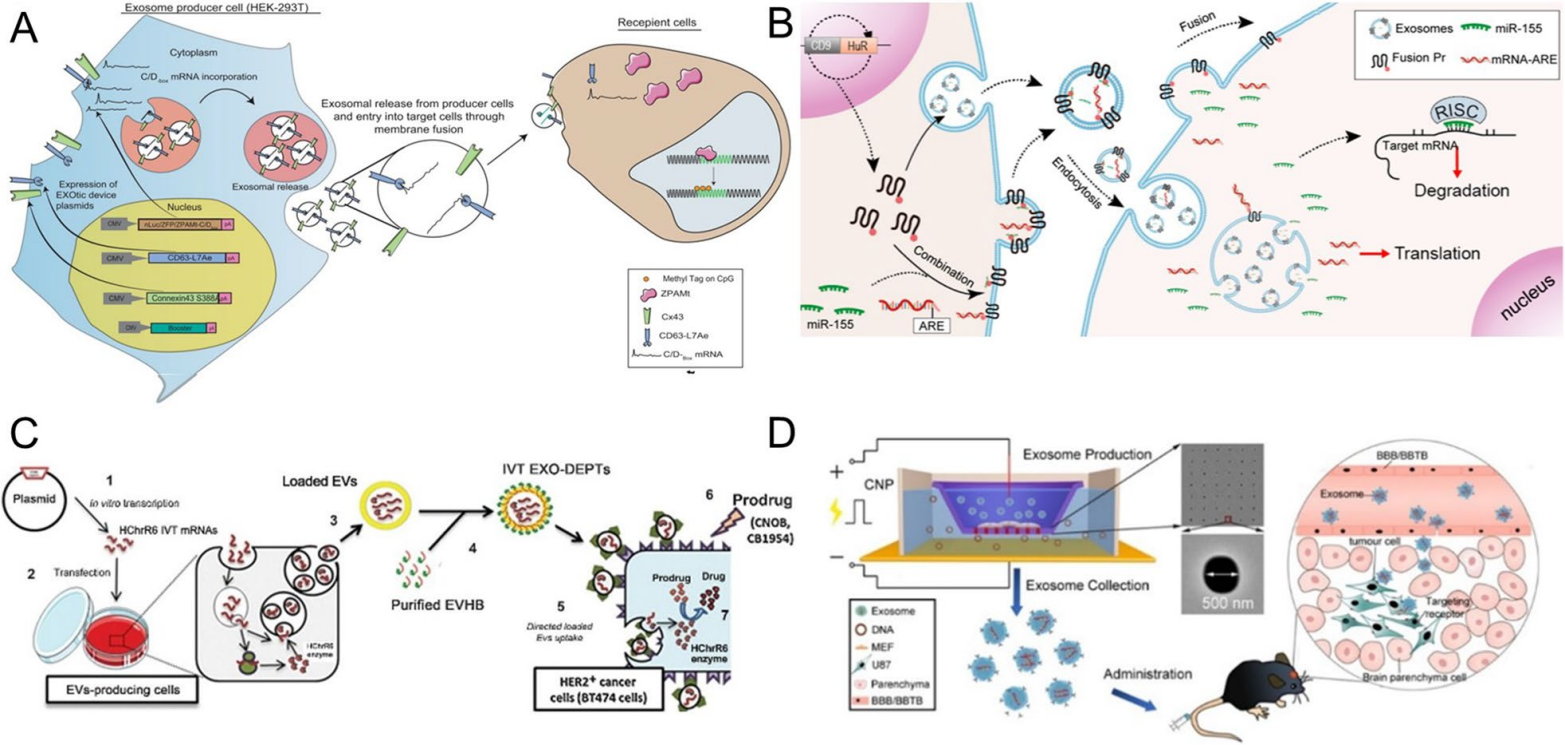
**A** An EXOsomal Transfer Into Cells (EXOtic) device recruits those mRNAs containing C/Dbox into budding exosomes [90]. **B** CD9-HuR functionalized exosomes deliver dCas9 mRNA to target gene C/ebpα related to cell proliferation and differentiation in liver [91]. **C** Exosomes loaded with HChrR6 mRNA for breast cancer therapy [93]. **D** a cellular nano perforation technology for producing a large number of exosomes containing therapeutic mRNAs [94]

As early as 2007, Valadi et al. [84] firstly found that exosomes were natural carriers of mRNA in mast cells. Subsequently, this phenomenon was also observed in many other cells [85–87]. However, the insertion of foreign mRNA into exosomes has been a challenge, because these electroporation- or chemical-based loading methods are not useful for packaging and delivering macromolecular mRNA via exosomes. Afterwards, Tsai et al. [88] reported that the exosome-liposome hybrid could efficiently transfect target cells with Antares2 mRNA. In the study, the purified mRNA was pre-incubated with polycationic lipid coating, and then mixed with equal amounts of purified exosomes. Based on this, the multiplexed mRNA COVID-19 vaccine was successfully developed. In addition, Kojima et al. [89] constructed an EXOsomal Transfer Into Cells (EXOtic) device, which transformed the way of sorting mRNA from natural but dynamic pathways to an engineering way. In this device, the archaea-derived L7Ae peptide (binding to the C/Dbox RNA structure) was fused with the exosomal marker protein CD63, recruiting those mRNAs containing C/Dbox into budding exosomes. And then exosomes carried these functional mRNAs into the cytoplasm of target cells (Fig. 4A). Recently, this method have also been adopted to package ZFP or ZPAMt mRNA with exosomes to inhibit HIV-1 transcription, inducing “blocking and locking” phenotypes in virusinfected cells [90]. In addition, Li et al. [91] fused the exosome membrane protein CD9 with RNA binding protein HuR to construct CD9-HuR functionalized exosomes, which has a strong ability to enrich specific RNAs (Fig. 4B). These functionalized exosomes were used to deliver dCas9 mRNA to target gene C/ebpα related to cell proliferation and differentiation in liver. And the expression of target gene was decreased by about 20-fold compared with free dCas9 mRNA. Furthermore, with the technical breakthrough of the exosome-loading mRNA method, this delivery system is gradually used to treat cancer.

#### Delivery of therapeutic mRNA

Wang et al. [92] applied exosomes to deliver HChrR6-encoding mRNA (generated by transfection of cells with the XPort/HChrR6 encoding plasmid) to the HER2+ve human breast cancer cells, which caused nearly complete growth arrest of the breast cancer cells. This was the first time that exosome-mediated exogenous mRNA delivery has gained a therapeutic advantage. Subsequently, Forterre et al. [93] have successfully utilized similar methods to treat HER2+ve human breast cancer cells. In the study, exosomes from HEK293 cells delivered functional HChrR6 mRNA to HER2+breast cancer cells, and when administered systemically along with prodrug CB1954, they arrested the growth of HER2+human breast cancer xenografts in athymic mice by prodrug activation (Fig. 4C). In another work, Usman et al. [33] treated leukemia cells with exosome from human red blood cells (RBCs) loaded with Cas9 mRNA and gRNA targeting the human miR-125b-2 (an oncogenic miRNA in leukemia) locus. The results indicated the expression of miR-125a and miR-125b decreased by 90–98% after 2 days of treatment.

In addition to the delivery of exogenous mRNA, endogenous functional mRNA has also been caught attention. Yang et al. [94] reported a cellular nano perforation technology for producing a large number of exosomes containing therapeutic mRNAs. Firstly, the plasmid DNA was transfected into various sources cells, and then the cells were stimulated with focal and transient electrical stimulation to promote the release of exosomes carrying the transcribed mRNA. Based on this, PTEN and CDX (CD47 cloning targeted peptide) plasmids were transferred into glioma cells to obtain a large number of targeted functional exosomes, which enhanced cell uptake, restored the expression of PTEN protein, inhibited tumor growth, and prolonged survival with a median survival of 45 days, compared with 31 days for non-functional exosomes (Fig. 4D). Encouragingly, NeoCura (a Chinese company of RNA precision medicine based on artificial intelligence) and MDimune lnc (a Korean company based on extracellular vesicle drug delivery platform) recently jointly developed mRNA therapy for cancer vaccine delivery based on exosomes [95]. Based on the above, exosome mediated mRNA delivery has promising potential for cancer treatment (Fig. 4).

### Exosomes-based miRNA delivery system

#### miRNA

miRNAs are a class of highly conserved single-stranded RNA with a length of 19–25 nucleotides, which are generally located in the non-coding region of the genome and do not encode proteins, but they play an important role in regulating gene expression [96, 97]. The specific sequence of the 3′ untranslated region (3′-UTR) of miRNA is completely or partially complementary to its targeted mRNA, leading to target degradation or translation inhibition, so as to negatively regulate the target protein expression. This process is also involved in the occurrence and development of tumors. For instance, several miRNAs, such as miR-149-5p, miR-29b and miR-34b, are poorly expressed in prostate cancer tissues [98]. There are some down-regulated miRNAs in bladder cancer, such as miR-145, miR-125b and miR-143, which even show anti-oncogenic properties; while some upregulated miRNAs, for instance, miR-17-5p, miR-20a, and miR-183, were oncogenic [99]. In NSCLC cells, miR-26, miR-21, miR-155 and miR-574-5p affected the progression of NSCLC through cell cycle regulation, escaping, apoptosis, metastasis regulation, etc. [100]. Besides endogenous miRNA, synthetic anti-miRNA oligonucleotides (AMOs) or miRNA mimics (miR mimics) have also been delivered into cells to suppress or enhance specific endogenous miRNAs’ function. Thus, regulating the expression of cancer-related genes through miRNA complementation is becoming a promising means of cancer treatment.

#### Loading methods for miRNA into exosomes

Recently, exosome based-miRNA therapy has developed more rapidly owing to its wide participation in gene regulation, small size, and easy to load. Electroporation is also used to load miRNA into exosomes. Studies have shown that each exosome was loaded with about 3000 miRNA molecules [101]. Table 2 also summarizes these studies of electroporation of miRNA into exosomes for therapy. In addition, miRNA could be loaded into exosomes by incubation at 37 °C [102]. However, the loading efficiency is not satisfactory, so this method is not often used. Besides, there are also commercial transfection reagents on the market, such as Exo-FectTM exosome transfection reagent, HiPerFect transfection reagent, Lipofectamine 2000 and 3000, which are used to load miRNA directly into exosomes (Table 2). Furthermore, in the case of heat shock, CaCl_2_ can mediate the transfection of miRNAs or their inhibitors into exosomes, and these RNAs have functional activity after transmission to recipient cells [44]. Additionally, another transfection method pre secretion of exosomes has also been proved to be effective. Trivedi et al. [103] introduced miRNA-125b into SK-LU-1 lung cancer cells using hyaluronic acid-polyethyleneimine (HA-PEI)/hyaluronic acid-polyethylene glycol (HA-PEG) combined nanoparticles as gene transfection agents, which successfully increased miRNA-125b expression in exosomes secreted by the lung cancer cells. Therefore, these available loading methods can be selected according to different requirments.

**Table 2:** The application of exosomes-based exogenous miRNA delivery system in cancer treatment in the last decade

Exosome	The source of exosome	Therapeutic cargo	Loading method	Target gene	Mechanisms	Cancer types (cell lines)	Effects	References
Engineering exosome (Apo-A1-modified exosome)	HEK293T cells	miR-26a	Electroporation	*CCNE2*, *CDK6*, *CCND2*	Down regulating of the expression levels of CCNE2, CCND2 and CDK6	Liver cancer (HepG2)	Decreasing the rates of cell migration and proliferation	[21]
Engineering exosome (GE11 peptide or EGF-modified exosome)	HEK293T cells	let-7a	HiPerFect transfection reagent	Unidentified or uncharacterized genes		Breast cancer (HCC70 HCC1954, MCF-7)	Inhibiting tumor development	[41]
Engineering exosome (magnetic molecules and L17E peptide- modified exosome)	Serum	dox, cholesterol-modified miR-21 inhibitor	Co-incubation	miR-21	Interfering with nuclear DNA activity and down regulating the expression of oncogenes *bcl-2*, *caspase-3* and *p-akt*	Human glioblastoma (U87), breast cancer (MDA-MB-231)	Inhibiting the growth of the tumors and alleviating side effects	[108]
Stem-cell-derived exosome	Human umbilical cord mesenchymal stromal cells	miR-145-5p	Exo-Fect™ exosome transfection reagent	*Smad3*	Activating the TGF-β/Smad3 pathways	Pancreatic ductal adenocarcinoma (Capan-1, CFPAC-1, BxPC-3, Panc-1)	Inhibiting cell proliferation and invasion and increasing apoptosis and cell cycle arrest	[109]
Stem-cell-derived exosome	Bone marrow mesenchymal stem cells	LNA-antimiR-142-3p	Electroporation	*APC*, *P2X7R*	Decreasing the levels of miR-142-3p and miR-150, and increasing the targeted regulation of *APC* and *P2X7R*	Breast cancer (MCF-7)	Reducing cell clonogenicity and tumorigenicity	[110]
Stem-cell-derived exosome	Human umbilical cord mesenchymal stem cells	miR-6785-5p mimic	Lipofectamine 2000 transfection reagent	*INHBA*	Inhibiting the expression of *INHBA*	Gastric cancer (SGC7901, MGC803)	Suppressing cell angiogenesis and metastasis	[111]
Stem-cell-derived exosome	Human bone marrow mesenchymal stem cells	miR-205 mimic	Lipofectamine 2000 transfection reagent	*RHPN2*	Inhibiting the expression of *RHPN2*	Prostate cancer (LNCaP)	inhibiting cell proliferation, invasion, and migration and promoting cell apoptosis	[112]
Stem-cell-derived exosome	Human umbilical cord mesenchymal stem cells	miR-139-5p mimic	Lipofectamine 2000 transfection reagent	*PRC1*	Inhibiting the expression of *PRC1*	Bladder cancer (T24, J82, UMUC3, 5637)	Impeding the cell proliferation, migration, and invasion potentials	[113]
cancer-associated fibroblasts	Cancer-associated fibroblasts	miR-3188 mimic	Lipofectamine 2000 transfection reagent	*BCL2*	Downregulating the expression of *BCL2*	Head and neck cancer (HN4, HN30)	Inhibiting cell proliferation, colony formation ability and G1 to S cell cycle transition	[114]
Cancer-associated fibroblasts	Cancer-associated fibroblasts	miR-320a mimic	Lipofectamine 2000 transfection reagent	*PBX3*	Suppressing the activation of the MAPK pathway	Hepatocellular carcinoma (MHCC97-H, SMMC-7721, Huh7)	Suppressing cell proliferation, migration and metastasis	[115]
Cancer-associated fibroblasts	Cancer-associated fibroblasts	miR-139 mimic	Lipofectamine 2000 transfection reagent	*MMP11*	Decreasing the expression of *MMP11*	Gastric cancer (N87, AGS)	Inhibiting cell growth and metastasis	[116]
Cancer-associated fibroblasts	Cancer-associated fibroblasts	miR-34 mimic	Lipofectamine 3000 transfection reagent	*AR*, *CCL22*, *CCND1*, *CCNE2*, *CDK4*, *CDK6*, *c-Met*, *E2F3*, *E2F5*, *HMGA2*, *LETK3*, MTA2, *N-Myc*, *PAR2*, *SFRS2*, *SIRT1*	Downregulating the expression of target genes	Gastric cancer (AGS, AZ521, MKN1, NUGC3)	Inhibiting cell proliferation and invasion	[117]
Exosome-liposome hybrid	Human ovarian cancer cells	miR497, triptolide	Liposome	*PI3K, AKT, mTOR*	activatingpi3k/AKT/mTOR signaling pathway	Ovarian cancer (SKOV3)	Signifcantly enhancing tumor cell apoptosis and overcoming chemoresistant ovarian cancer	[118]
Engineering exosome (Her2-LAMP2-modified exosome)	HEK293T cells	miR-21 inhibitor, 5-FU	Electroporation	miR-21	Downregulating miR-21 and rescuing *PTEN* and *hMSH2* expressions	Colon cancer (HCT-116^5FR^)	Reversing drug resistance and significantly enhancing the cytotoxicity in 5-FU-resistant colon cancer cells	[107]
	HEK293T cells	Let7c-5p	Lipofectamine RNAiMAX reagent	*HMGA2*, *c-Myc*	Downregulating the expression of *HMGA2* and *c-Myc*	Breast cancer (MDA-MB-231)	Inhibiting cell proliferation and migration	[119]
Stem-cell-derived exosome	Bone marrow-derived mesenchymal stem cells	LNA anti-miR-142-3p	Electroporation	*APC*, *P2X7R*	Suppressing the expression level of miR-142-3p and miR-150 and increasing the transcription of the regulatory target genes, *APC* and *P2X7R*	Breast cancer (4T1)	Decreasing cell proliferation	[120]
Stem-cell-derived exosome	Human umbilical cord mesenchymal stem cells	miR-375 mimic	Transfection	*ENAH*	Suppressing *ENAH* expression	Esophageal squamous cell carcinoma (KYSE70, ECA109, EC9706)	Inhibiting cell proliferation, invasion, migration, tumorsphere formation, and promoting apoptosis	[121]
Engineering exosome (TAT peptide-modified exosome)	A549 cells	miR-449a	TAT-TAR interaction	*Bcl-2*	Inhibiting the expression of apoptosis inhibitor protein Bcl-2	Non-small cell lung cancer (A549)	Inhibiting cell proliferation and promoting cell apoptosis	[43]
Engineering exosome (T7 peptide-modified exosome)	HEK293T cells	miR-21 antisense oligonucleotides	Electroporation	*miR-21*	Reducing of miR-21 and inducing the expression of PDCD4 and PTEN	Glioblastoma (C6)	Resulting in a reduction of tumor sizes	[106]
Cancer-cell-derived exosome	HT-29 and SW480 cells	miR-375-3p mimic	Modified calcium chloride method	*ZEB1*	Reducing the expression of β-catenin, vimentin, ZEB1, and snail significantly increasing the expression of E- cadherin in EMT process	Colon cancer (HT-29, SW480)	Reversing EMT process and inhibiting cell invasion and migration	[44]
	FHC cells	miR-128-3p mimic	Electroporation	Bmi1, MRP5	Suppressing *Bmi1* and *MRP5* expression	Oxaliplatin-resistant colorectal cancer (HCT116OxR, HT29OxR)	Enhancing cell chemosensitivity	[122]
	HEK293T cells	miRNA-497 mimic	Transfection	*YAP1*, *HDGF*, *CCNE1*, *VEGF-A*	Suppressing *YAP1*, *HDGF*, *CCNE1*, *VEGF-A* expression	Non-small cell lung cancer (A549)	Inhibiting the tumor growth and angiogenesis	[123]
	SCC084 cisplatin- resistant strain	miR-30a mimic	Lipofectamine RNAiMAX	*Beclin1*	A concomitant decrease in *Beclin1* and *Bcl2* expression	Oral squamous cell carcinoma (SCC084)	Regaining sensitivity of the cisplatin-resistant OSCC cells	[124]
Cancer-cell-derived exosome	Oral cancer patients and oral squamous cell carcinoma (OSCC)	miR-155 mimic	Lipofectamine RNAimax	*FOXO3a*	Modulation of EMT pathway and downregulation of FOXO3a	Oral cancer (SCC131)	Conferring cisplatin resistance in OSCC	[104]
	Parental and cisplatin-resistant human OSCC cell lines	miR-155 inhibitor	Modified calcium chloride transfection method	*FOXO3a*	Upregulation of FOXO3a and induction of the mesenchymal-to-epithelial transition	Oral squamous cell carcinoma (UPCI-SCC-131)	Reversing chemoresistance in oral cancer	[46]
	Cisplatin-resistant OSCC cells	Anti-miR-21	Lipofectamine 3000	*PTEN*, *PDCD4*	Downregulating the expression of *PTEN* and *PDCD4*	Oral squamous cell carcinoma (HSC-3, SCC-9)	Inducing cisplatin resistance of OSCC cells	[125]
	HEK293T cells	Anti-miR-214	Lipofectamine 2000 transfection reagent	miR-214	Downregulation of miR-214 and overexpression of possible target proteins (PARP9, XRCC, LIN28B)	Gastric cancer (SGC7901)	REVERSING chemoresistance and repressing tumor growth	[126]
	HEK293T cells	miR-199a-3p mimic	Lipofectamine 2000 transfection reagent	*ZEB1*, *MTOR*, *DNMT3A*	Down-regulation of underlying target proteins (*ZEB1*, *MTOR*, *DNMT3A*)	Hepatocellular carcinoma (Huh-7)	Reversing chemoresistance to cisplatin in hepatocellular carcinoma	[127]
	Doxorubicin-resistant gastric cancer SGC7901/ADR cell	miR-501 inhibitor	Lipofectamine 2000 transfection reagent	*miR-501*	Inducing downregulation of BLID, inactivating of caspase-9/-3 and phosphorylation of Akt	Gastric cancer (SGC7901)	Being sensitive to doxorubicin and attenuating proliferation, migration and invasion and increasing apoptosis	[105]

#### Delivery of therapeutic miRNA

**Fig. 5:**
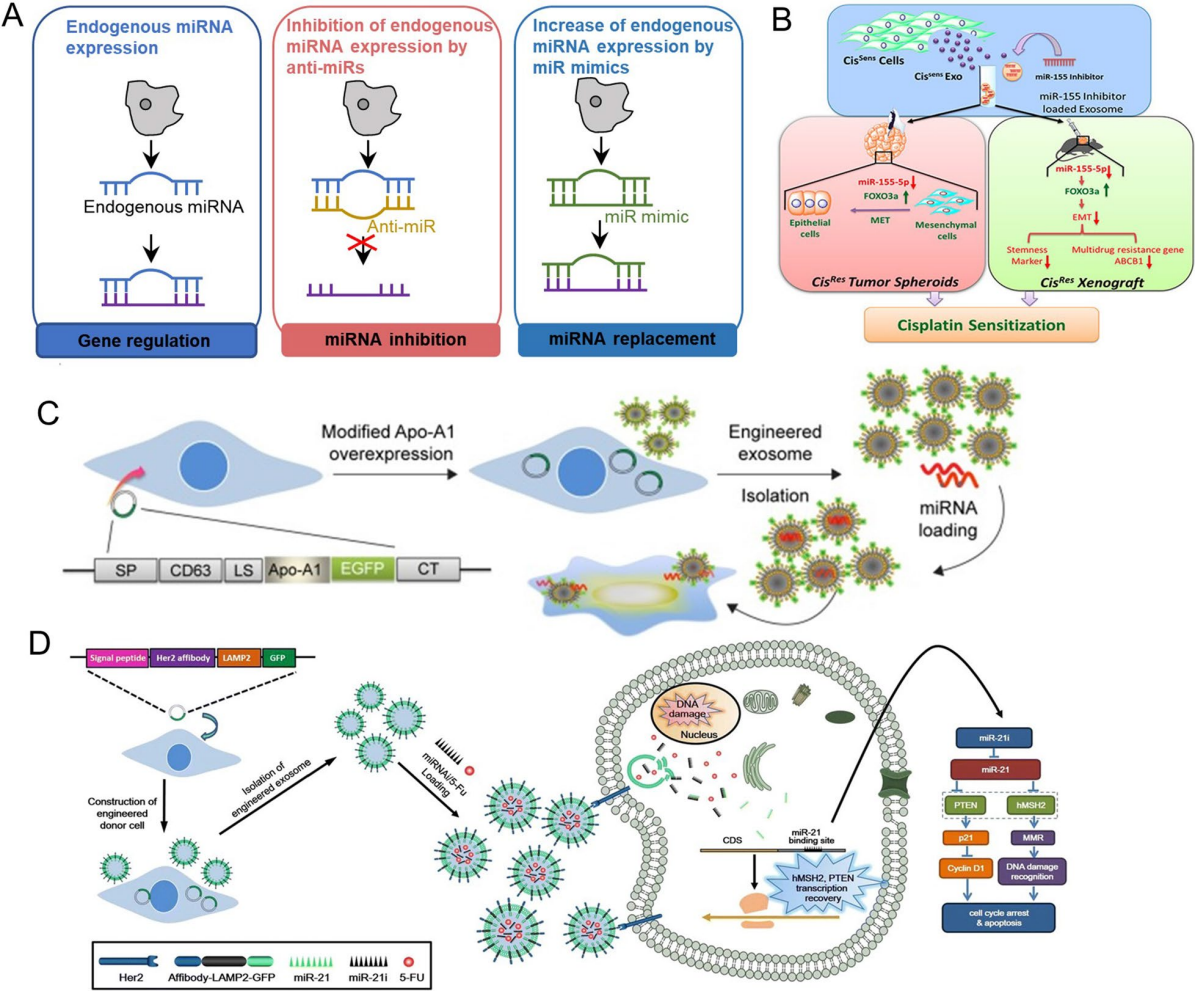
**A** Different therapeutic forms of miRNA. **B** Exosome mediated miR-155 inhibitor delivery to treat cisplatin-resistance oral squamous cell carcinoma [104]. **C** Apo-A1-modified exosomes loaded miR-26a selectively bound to HepG2 cells via the SR-B1 receptor-mediated endocytosis [21]. **D** Engineered exosome packaged miR-21i and chemotherapeutics 5-Fluorouracil (5-FU) to target 5-FU-resistant colorectal cancer cell [107]

miRNA-based therapy is divided into two forms: miRNA replacement or inhibition (Fig. 5A). The former aims to introduce exogenous miRNAs (miR mimics) known to promote tumor inhibition. The latter provides specific miRNA inhibitor or AMOs to inhibit tumor promoting miRNA (oncomiR). Based on the different therapeutic requirements, a large number of successful cases have been reported. For example, as a tumor suppressor, miR-375 is negatively associated with epithelial-mesenchymal transition (EMT) in cancer patients. To increase its expression and reverse EMT process, Rezaei et al. [44] used tumor-derived exosomes to deliver miR-375 mimic, resulting in the inhibition of the migration and invasion abilities of colon cancer cells. In addition, miR-155 overexpression can enhance the invasive and chemoresistance of oral squamous cell carcinoma (OSCC) cells. To decrease miR-155 expression, Kirave et al. [104] introduced exosome as a carrier and miR-155 inhibitor as therapeutic agent to treat cisplatin-resistance OSCC, and the results revealed that exosomes loaded miR-155 inhibitor could reverse chemoresistance in oral cancer through upregulating the expression of *FOXO3a* and inducing the EMT transition (Fig. 5B). Similarly, exosome mediated miR-501 inhibitor delivery into doxorubicin (dox)-resistant gastric cancer cell, resulting in inhibiting the expression of miR-501 and makeing the cells sensitive to dox [105]. Although exosome based-miRNA has some effect in the tumor treatment, the therapeutic effect needs to be improved due to its targeting.

Genetically modified exosome can target tumor cells through binding functional ligands modified on exosomal surface to overexpressed receptors on the tumor surface, so as to transfer more miRNA to tumor cells and further enhance the therapeutic effect. For example, Liang et al. [21] reported that the Apo-A1-modified exosomes loaded miR-26a (Apo-Exo/miR-26a) selectively bound to HepG2 cells via the SR-B1 receptor-mediated endocytosis. The results revealed that compared with the HepG2 cells incubated with exosome-loaded miR-26a, those with Apo-Exo/miR-26a could upregulate miR-26a expression about threefold, and downregulate key cyclins CCNE2 and CDK6 expression about onefold, and the inhibition of cell migration was twofold as high (Fig. 5C). Ohno et al. [41] revealed that modified exosomes with the GE11 peptide on their surfaces delivered let-7a miRNA specifically to xenograft breast cancer tissue in RAG2^–/–^ mice, significantly inhibiting tumor development in vivo through upregulating the let-7a expression about 1000-fold and downregulating target gene *HMGA2* about fivefold. Similarly, exosome surface were modified with target peptide transcriptional transactivator (TAT) protein and T7 respectively to deliver different miRNA to target tumor cells, which obviously inhibiting the tumor occurrence and development [43]^43^. Although the efficacy of this single gene therapy can be improved by strengthening the targeting of exosomes, due to the limitation of monotherapy, it is necessary to cooperate with other therapies to further improve the therapeutic effect. 

Recently, it has been demonstrated that miRNAs and chemotherapeutics can be co encapsulated within engineered exosome and achieve more excellent anti-tumor effect. Liang et al. [107] integrated the fusion protein Her2-LAMP2 into the surface of exosomes to make exosome target EGFR receptor. And then engineered exosome packaged miR-21i and chemotherapeutics 5-Fluorouracil (5-FU) (THLG-EXO/5-FU/miR-21i) to target 5-FU-resistant HCT-116 colorectal cancer cell (HCT-116^5−FR^) through EGFR receptor-mediated endocytosis. The results revealed that the apoptotic proportion and proliferation inhibition rate of THLG-EXO/5-FU/miR-21i-treated HCT-1165^FR^ cells increased by about 3.5-fold and fivefold respectively compared with that of THLG-EXO/miR-21i-treated cells (Fig. 5D). In addition, Zhan et al. [108] designed the exosome: (1) safe and sufficient blood exosomes; (2) binding the ligand-coupled superparamagnetic nanoparticles to the specific membrane proteins of exosome to achieve the separation, purification and tumor magnetic-targeting; (3) co-loading hydrophobic drugs dox and cholesterol-modified miR-21i to enhance the therapeutic effectiveness; (4) binding L17E peptide to promote the cytosolic release of encapsulated cargos. The engineered exosomes (D-Exos/miR21i-L17E) that met the above four requirements could be highly enriched in tumor targets. The results revealed that compared with Exo/miR21i-L17E groups, antitumor effect in vivo decreased two folds at the 18th day after administration in the D-Exos/miR21i-L17E group. Based on the above, the excellent antitumor could achive through combined therapy. The related research have been summarized in Table 2 in detail.

### Exosomes-based siRNA delivery system

#### siRNA

siRNA is another class of double stranded DNA (dsRNA) with a length of about 25 bp, which could completely complementary to the targeted mRNA, resulting in gene silencing [128–131]. The mechanism is that endogenous dsRNA is recognized by ribonuclease protein Dicer, which cleaves the dsRNA into 21 to 23 bp with 2-nucleotide overhanging at 3′ ends. These cleavage products, named siRNAs, consist of a passenger and guide strands. After binding to the RNA-Induced Silencing Complex (RISC), the guide chain is guided to the target mRNA and cleaved into small fragments by the cleavage enzyme argonaute-2, which is located between bases 10 and 11 at the 5′ ends of the siRNA guide chain [132, 133]. Based on the above, siRNA has the potential to treat a variety of diseases by regulated the expression of target mRNA. Recently, FDA approved the Patisiran (siRNA is delivered to hepatocytes as a lipid complex) and Givosiran (siRNA is coupled to a GalNAc ligand that makes salivary glycoprotein receptors-mediated targeted delivery to hepatocytes) siRNA drugs, marking the beginning of the era of RNA interference (RNAi) therapy [134, 135].However, the successful therapy requires the safe and efficient delivery of siRNA into the cytoplasm to play an interference function.

#### Loading method for siRNA into exosomes

The concept of delivering siRNA using exosomes was first confirmed by Alvarez-Erviti et al. [136], who electroporating exogenous siRNA into exosomes for delivery both in vitro and in vivo, resulting in the knockdown of the specific gene *BACE1*. Similarly, some studies loaded siRNA into exosomes by electroporation (Table 3). Furthermore, Wahlgren et al. [137] delivered therapeutic siRNA into exosomes from peripheral blood by electroporation. And the effects of exosome concentration, siRNA concentration, and electroporation parameters on electroporation efficiency were studied. The results revealed that the changes of siRNA and capacitance had no effect on the electroporation efficiency, and when the concentration of exosomes was in the range of 0.25-1 mg/ml, the electroporation efficiency was the highest. In addition, like miRNA, there are also some commercial transfection reagents for transfecting siRNA into exosomes, such as Lipo2000, Lipo3000, Exo-fect Exosome Transfection Reagent, etc. (Table 3). Aqil et al. [138] loaded siRNA into milk exosomes through the Exo-fect Exosome Transfection Reagent, and the loading efficiency was about sixfold higher than that of electroporation. Besides, siRNA loaded into exosomes by sonication could be delivered to breast cancer cells, resulting in a 50% knockdown of an oncogene [139]. Exosomes by sonication induced less siRNA aggregation than electroporation [140]; However, the number of siRNAs entering recipient cells through exosomes is still limited. Therefore, sonication parameters need to be optimized to improve loading efficiency. As the loading methods mature, it is gradually applied to cancer therapy.

**Table 3:** The application of exosomes-based siRNA delivery system in cancer treatment in the last decade

Exosome	The source of exosome	Therapeutic cargo	Loading method	Target gene	Mechanisms	Cancer types (cell lines)	Effects	References
Engineering exosome (tLyp-1-modified exosome)	HEK293T cells	siR1, siR2, siR3	Electroporation	*SOX2*	Knock-down the target gene expression	Non-small cell lung cancer (A549)	Reducing the stemness of cancer stem cells	[151]
Cancer-cell-derived exosome	Autologous breast cancer cells	siS100A4	Incubation and extrusion method	*S100A4*	Down-regulate the expression of S100A4	Triple-negative breast cancer (4T1)	Inhibiting the growth of malignant breast cancer cells	[152]
	HEK293T cells	si–c-Met	Lipofectamine 2000 transfection reagent	*c-Met*	Inhibiting the expression of c-Met	Gastric cancer (SGC7901)	Reversing the drug resistance of gastric cancer cells in vitro, and significantly inhibiting the tumor growth	[146]
Engineering exosome (FA-displaying exosome)	HEK293T cells	Survivin siRNA	ExoFect exosomes transfection reagent	*survivin*	Knockdown the expression of survivin	Human oral epidermal carcinoma (KB)	Inhibiting tumor growth	[139]
Stem-cell-derived exosome	HEK293 cells, mesenchymal stem cell	PLK-1 siRNA	Electroporation	*PLK-1*	Knockdown of PLK-1 mRNA and protein	Bladder cancer (UMUC3, SW780)	Inhibiting the bladder cancer cell proliferation	[142]
Engineering exosome (DARPin G3- modified exosome)	HEK293T cells	TPD52 siRNA	Electroporation	*TPD52*	Binding specifically to HER2/Neu and siRNA molecules against TPD52 gene	Breast cancer (MDA-MB-231)	Inhibiting tumor growth	[153]
	Normal fibroblast-like mesenchymal cells	KRAS siRNA	Electroporation	*KRAS*^*G12D*^	Reducing *KRAS*^G12D^ mRNA levels and phosphorylated-ERK protein levels	Pancreatic cancer (MIA-PaCa-2, Capan-1)	Inhibiting tumor metastasis and increasing overall mouse survival	[154]
	HEK293T cells	HGF siRNA	Lipofectamine 2000 transfection reagent	*HGF*	Activating the HGF/c–Met signaling pathway	Gastric cancer (SGC‐7901)	Suppressing tumor growth and angiogenesis	[155]
Cancer-cell-derived exosome	Cancer-associated fibroblasts	LINC00355 siRNA	Lipofectamine 2000 transfection reagent	*LINC00355*	Decreasing the expression of LINC00355	Bladder cancer (T24, 5367)	Repressing cell proliferation and invasion	[156]
Cancer-cell-derived exosome	Breast cancer	MALAT1 siRNA	Lipofectamine 2000 transfection reagent	*MALAT1*	Down-regulating the expression of MALAT1	Breast cancer (MCF-7, MDA-MB-231, MDA-MB-435S)	Suppressing cell proliferation	[157]
Cancer-cell-derived exosome	PANC-1 cells	PAK4 siRNA	Electroporation	*PAK4*	Down-regulating the expression of PAK4	Pancreatic cancer (PANC-1)	Inhibiting tumor growth and increasing mice survival	[158]
	Human skin-derived fibroblasts(NB1RGB cells)	LCP1 siRNA	Electroporation	*LCP1*	Suppressing LCP1 expression	Oral cancer (HSC-2, HSC-3, HSC-3-M3, HSC-4, Sa3, Ca9-22, KOSC-2, SAS, Ho-1-u-1, Ho-1-N-1, SAS-H1)	Suppressing the oncogenic activity of cancer cells	[159]
	HEK293T cells	TRPP2 siRNA	Incubation	*TRPP2*	Suppressing TRPP2 protein expression levels	Human pharyngeal squamous cell carcinoma (FaDu)	Inhibiting migration, invasion and the EMT of cancer cells	[160]
Cancer-cell-derived exosome	MCF-7, MCF-7/ADR cells	CD44 siRNA	Electroporation	*CD44*	Suppressing CD44 expression	Breast cancer (MCF-7/ADR)	Reducing cell proliferation and enhancing susceptibility to DOX	[161]
	MCF10A cells	CDK4 siRNA	Electroporation	*CDK4*	Downregulating the CDK4 mRNA and protein expression	Breast cancer (MCF-7)	Inhibiting tumor growth	[162]
	HeLa cells	RAD51 siRNA, RAD52 siRNA	Electroporation	*RAD51*, *RAD52*	Downregulating RAD51/RAD52 expression	Human cervical carcinoma (HT1080)	Resulting in apoptosis of the tumor cells	[40]
Engineering exosome (iRGD peptide-modified exosome)	HEK293T cells	KRAS siRNA	Lipofectamine 2000 transfection reagent	*KRAS*	Silencing KRAS gene expression	Lung cancer (A549)	Inhibiting tumor growth	[163]
Stem-cell-derived exosome	Bone-marrow-derived mesenchymal stem cells	GRP78 siRNA	Lipofectamine 2000 transfection reagent	*GRP78*	Inhibiting the expression of GRP78	Hepatocellular carcinoma (HepG2, PLC)	Inhibiting the growth and invasion of the cancer cells	[145]
Stem-cell-derived exosome	Bone marrow mesenchymal stem cell	Galectin-9 siRNA	Electroporation	*galectin-9*	tumor-suppressive macrophage polarization, cytotoxic T lymphocytes recruitment and Tregs downregulation	Pancreatic ductal adenocarcinoma (PANC-02)	Eliciting anti-tumor immunity	[100]
Engineering exosome (cRGD peptide-modified exosome)	RAW 264.7 cells	FGL1 siRNA, TGF-β1 siRNA	Exo-fect Exosome Transfection Reagent	*FGL1*, *TGF-β1*	Blocking immune checkpoint FGL1	Colorectal cancer(MC38)	An increased number of tumor infiltration CD8 + T cells, a decreased number of immunosuppressive cells, a significant anti-tumor effect	[150]
	HEK293 cells	SCD-1 siRNA	Electroporation	*SCD-1*	Regulating of fatty acids metabolism and increasing ROS level	Anaplastic thyroid carcinoma (Hth-7)	Inhibiting cellular proliferation and promoting cellular apoptosis	[164]
Engineering exosome (iRGD peptide-modified exosome)	HEK-293 T cells	CPT1A siRNA	Lipofectamine 2000 transfection reagent	*CPT1A*	Regulating fatty acid oxidation	Colon cancer (HCT116, sw480)	Reversing oxaliplatin resistance and inhibiting tumor growth	[147]
	Natural killer cells NK92MI	BCL-2 siRNA	Co-incubation	*BCL-2*	Inhibiting the expression of BCL2	Breast cancer (MCF-7, SKBR3, T-47D, MDA-MB-231)	Enhancing cancer cell’ intrinsic apoptosis	[141]
Engineering exosome (E3 aptamer- modified exosome)	HEK293T cells	SIRT6 siRNA	Electroporation	*SIRT6*	Inhibiting the expression of SIRT6	Prostate cancer (C42B, DU145)	Inhibiting tumor growth and metastasis	[165]
Engineering exosome (EGFR RNA aptamer-modified exosome)	HEK293T cells	Survivin siRNA	ExoFect exosome transfection	*survivin*	Knockdown the expression of survivin	Non-small-cell lung cancer (A549)	Leading to potent gene knockdown, chemotherapy sensitization, and tumor regression	[166]
Engineering exosome (RNA nanotechnology- modified exosome)	HEK293T cells	Survivin siRNA		*survivin*	Knockdown the expression of survivin	Prostate cancer breast cancer colorectal cancer	Inhibiting tumor regression	[143]

#### Delivery of therapeutic siRNA

**Fig. 6:**
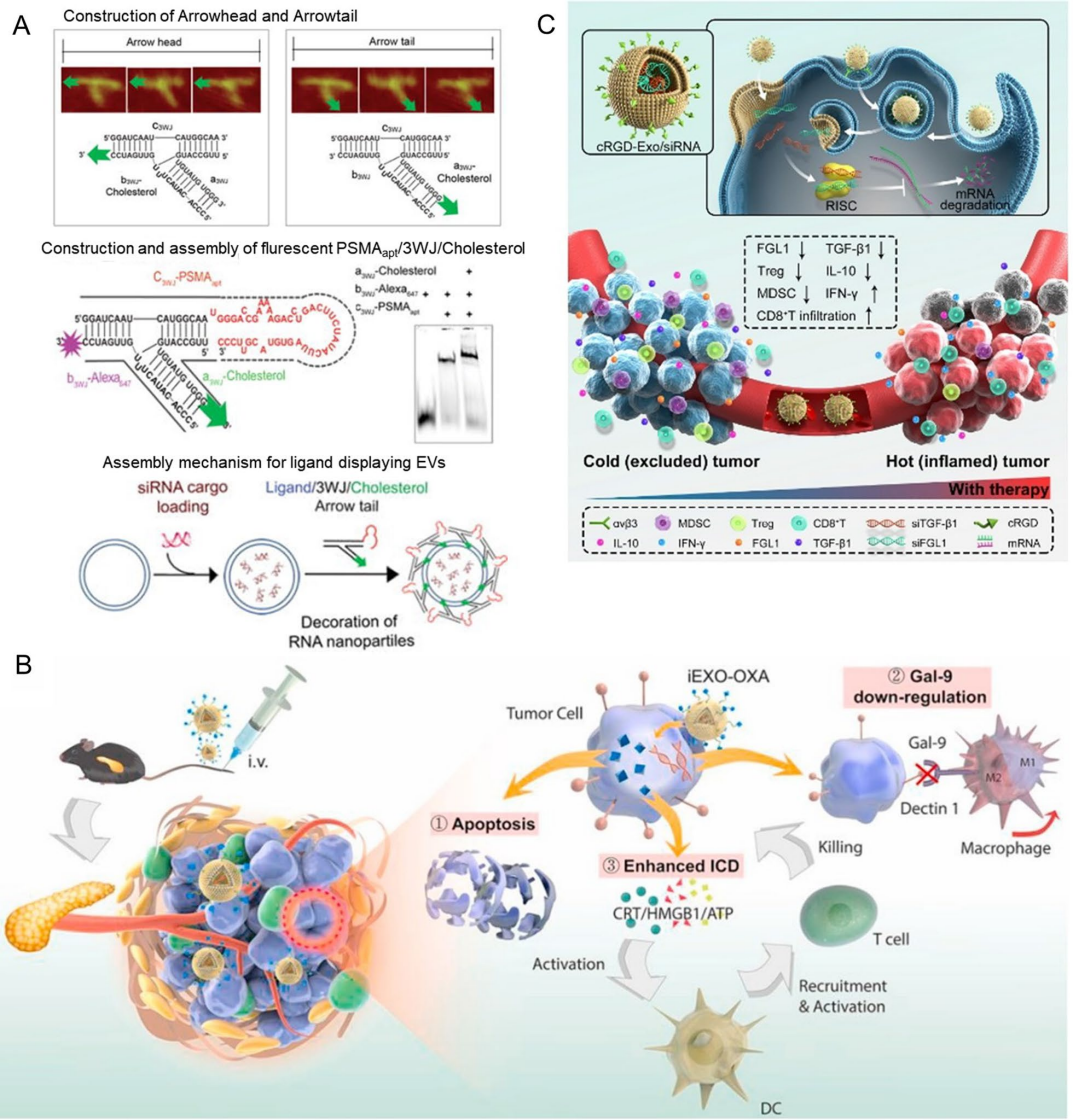
**A** RNA nanoparticle and RNA nanoparticles-modified exosome [143]. **B** Exosomes co-delivery chemotherapy drugs oxaliplatin (OXA) and nucleic acid drugs gal-9 siRNA to enhancing immunotherapy and reprogramming tumor microenvironment (TME) [100]. **C** cRGD-modified exosome with high siFGL1 and siTGF-β1 loading efficiency to realize the co-silence of FGL1 and TGF-β1 to to block immune checkpoints and simultaneously regulate TME [150]

As we all know, cancer progression is related to the up-regulation of anti apoptotic protein such as BCL-2, PLK1, KRAS, survivin protein that initiates cell mitosis, and cell growth factor. siRNA exosomal therapy targeting tumor cells has been been committed to downregulating these oncogenes expression to inhibit cell proliferation and migration. Kaban et al. [141] loaded BCL-2 siRNA into natural killer (NK) cell-derived exosomes to treat ER + breast cancer, leading to enhanced apoptosis in breast cancer cells. Similarly, exosomes mediated the delivery of PLK-1 siRNA into bladder cancer cells, promoting cell apoptosis through silencing PLK-1 expression [142]. Additionally, Pi et al. [143] designed RNA nanoparticles-modified exosome to simultaneously target three cancer cells (breast cancer, prostatic cancer and colorectal cancer). In the structure of the RNA nanoparticle, the pRNA of phage phi29 (an RNA molecule with transport function) was extended into an arrow shape and connected with an RNA ligand (used to target and bind specific overexpressed receptors in tumor cells) and added the fluorescent dye alexa647 for imaging. The three RNA ligands were designed: prostate cancer specific membrane antigen RNA ligand, epidermal growth factor RNA ligand and folic acid ligand. And then this modified exosomes were used to deliver survivin siRNA. The results revealed that compared with the control (injection PBS), during the entire treatment period, the growth of breast and prostate cancer cells treated with the above exosome loaded with survivin siRNA was completely inhibited,the growth inhibition rate of colorectal cancer cell treated with the functional exosome increased onefold (Fig. 6A).

As mentioned above, exosome mediated siRNA delivery can effectively inhibit the proliferation and migration of cancer cells. However, drug resistance is another major challenge in cancer treatment. Generally speaking, overexpression of chemotherapy resistance-associated proteins caused drug resistance in cancer cells [144]. Utilizing siRNA to overcome drug resistance has been widely reported. For example, Li et al. [145] used exosomes from bone marrow mesenchymal stem cells (BM-MSC) to deliver siRNA against Grp78 (overexpression in hepatocellular carcinoma and could promote the drug resistance to Sorafenib) in Sorafenib-sensitive hepatocellular carcinoma cells, leading to sorafenib-resistant cancer cells’ sensitivity sorafenib and the reversal of drug resistance. Similarly, Zhang et al. [146] reported that si-c-Met delivered by exosome showed a better inhibitory effect on the expression of c-Met (an essential role in drug resistance of various tumors) and significantly enhanced drug sensitivity. In addition, fatty acid oxidation (FAO) plays a crucial role in drug resistance of cancer cells. Carnitine palmitoyltransferase 1A (CPT1A), a key enzyme of FAO, is widely considered as an emerging therapeutic target. Lin et al. [147] utilized iRGD-modified exosomes to specifically deliver siCPT1A into colon cancer cells to suppress FAO, which have reversed the sensitivity of drug-resistant colon cancer cells to oxaliplatin. The above methods can effectively alleviate the drug resistance of cancer cells and provide new ideas for cancer treatment.

In addition, cancer immunotherapy utilizes the patient’s immune system to identify and destroy cancer cells, which is a specific protective strategy for cancer treatment [148]. Athough immune cells are common in the tumor microenvironment (TME), accounting for about 50% of the stromal cell components, only a few are anti-tumor effector cells, which may be responsible for the immune escape of tumor cells [149]. Thus, it is necessary for tumor immunotherapy to target TME and/or immune checkpoints. For example, Zhou et al. [100] designed a bio-platform targeting pancreatic ductal adenocarcinoma (PDAC) to enhance immunotherapy and reprogram TME. In this platform, exosomes derived from BM-MSCs as carrier co-delivered oxaliplatin (OXA) and gal-9 siRNA. Among them, OXA could both kill tumors and induce immunogenic cell apoptosis. siRNA interfered with the galectin-9 synthesis in tumor cells to reduce the transformation of macrophage M1. Compared with the chemotherapy or gene therapy alone, this combination treatment produced synergetic effects that affects cellular crosstalk in vivo, leading to overall change in TME, so as to further improve the antitumor efficacy, and the inhibition rate of cell growth was about twice higher (Fig. 6B). Furthermore, Pei et al. [150] established a cRGD-modified exosome with fibrinogen-like protein 1 (FGL1, an important immune checkpoint) siRNA and transforming growth factor-β (TGF-β1, an immunosuppressive cytokine in TME) siRNA (cRGD-Exo/siMix) to co-silence of FGL1 and TGF-β1. The results revealed that FGL1 expression was inhibited, which activated T cell recognition. Meanwhile, TGF-β1 expressiom was also silenced, which disaired the immunosuppressive microenvironment of tumor and promoted the infiltration of immune cells (Fig. 6C).

In general, exosome mediate the delivery of siRNA to silence the expression of key genes related to cell proliferation, drug resistance, immune checkpoints and TME, which will inhibit the development of tumor. Additionally, the simultaneous use of several siRNAs, and siRNA combined with chemotherapeutic drugs will achieve synergistic effects. The application of exosomes carrying siRNA for cancer treatment are summarized in Table 3 in detail. These methods will provide a reference for cancer treatment by using siRNA.

### Exosomes-based circRNAs delivery system

#### circRNAs

circRNAs are covalently closed RNA molecules without 5′ caps and 3′ tails, generated by a process of back-splicing [167]. The length ranges from hundreds to thousands of nucleotides, and they are highly abundant in eukaryotes [168]. The circRNAs play important roles in the occurrence and development of human diseases, especially cancer, which can regulate multiple cancer-related biological processes. The main mechanisms are as follows [169]: (a) acting as miRNA proton sponges: circRNA competitively binds miRNA to regulate the expression of miRNA and its target genes; (b) regulating gene transcription: exon–intron circRNAs (EIciRNAs) interact with U1 small nuclear ribonucleoprotein (snRNP) to form the EIciRNAs-U1 snRNP complex, which binds to polymerase II (Pol II) to regulate the promoter region of host gene transcription; (c) binding with protein: circRNAs act as protein sponges or baits to regulate gene expression; (d) encoding small functional peptides: circRNAs have ribosome binding sites and stable open reading frame (ORF), which can encode corresponding peptides. For example, CiRS‐7, one of the most famous circRNAs, acts as more than 70 conventional miR-7-binding sites and modulates the expression of multiple cancer-related genes [170, 171]. circRNA_FoxO3 can be used as a protein scaffold of MDM2 and p53 to induce p53 degradation, which can induce cancer cell apoptosis [172]. circRNA_SHPRH encodes protein SHPRH-146aa, which function as a bait to protect SHPRH protein from ubiquitination through DTL mediated degradation, so as to inhibit glioma occurrence [173]. Although the circRNAs have been served as one of the most promising biomolecules for cancer therapy, their delivery efficiency is often limited by the selected delivery system.

#### Delivery of therapeutic circRNA

circRNA naturally carried by exosomes has been widely developed for cancer treatment, which greatly improve its therapeutic effect owing to the high delivery efficiency. For example, Xue et al. [174] reported that exosomal circRNA_100284 acted as a sponge of miR-217, inhibiting cell proliferation by inducing a G2/M phase arrest in the cell cycle and targeting enhancer of zeste homolog (EZH) in various cancers. Chen et al. [175] introduced that exosomal circ-0051443 suppressed the hepatocellular carcinoma progression through competitive bounding to miR-331-3p. However, exosome-mediated exogenous circRNA delivery also faces some challenges. For one thing, the special circular structure of circRNA leads to the low circular efficiency. For another, macromolecular circRNAs also face the same problem as the large-size mRNA discussed above, which is difficult to load into the exosomes. To solve these problems, Yu et al. [176] constructed the target circRNA_DYM coding DNA into the lentivirus expression vector and then combined RVG-Lamp2b plasmid to transfected them into the HEK293T cells, and the engeneered exosome stably overexpressing the target circRNA_DYM (RVG-circDYM-EX) were secreted. This not only made the circRNA correctly and efficiently cyclized, but also could be easily loaded into the exosomes. The RVG-circDYM-EX was delivered to the brain to attenuate astrocyte disfunction induced by chronic unpredictable stress through binding to the transcription factor 1 (TAF1) and downregulating multiple downstream genes (*Trpm6*, *Cyp39al*). Similarly, Yang et al. [177] obtained the engeneered exosome modified with RVG-Lamp2b and loaded with circRNA_ SCMH1 and successfully transported them to the brain. The results revealed that the delivery system promoted functional recovery of rodent and non-human primate ischemic stroke models through binding to the methyl-CpG binding protein 2 and upregulating the expression of the target genes (*Mobp*, *Igfbp3*, *Fxyd1* and *Prodh*).

In conclusion, the circular structure of circRNA can not only prevent being degraded and improve the expression time and amount of circRNA, but also be administered repeatedly, which makes it one of the emerging nucleic acid drugs. Some natural exosomal circRNA can play an important role in cancer therapy. And exogenous circular RNA can also be cyclized efficiently by constructing related lentivirus vectors, and can be loaded into exsome through transfecting the vectors into the target cells, which will provide references for the application of this system in cancer.

### Exosomes-based other nucleic acids delivery system

#### Other nucleic acids

Other nucleic acid drugs, including long noncoding RNA (lncRNA), short hairpin RNA (shRNA), aptamer, etc., have been also introduced into cancer therapy. LncRNA, an RNA family with many members, has a length of over 200 bp and cannot be transformed into protein. Although it does not have the function of traditional RNA, it can regulate the activity of transcription factors [178, 179]. Moreover, some lncRNAs play a curical role in tumor proliferation, apoptosis, diffusion, and homeostasis maintenance [180–182]. For shRNA, structurally, it is more similar to miRNA, and both of them are local double-stranded RNA formed by hairpin structure [183]; Functionally, it is closer to siRNA, which is cleaved by the Dicer to form siRNA, and then performs interference through the siRNA pathway [184]. The shRNA is also a critical effector molecule in RNAi technology, and it could induce target mRNA degradation [185]. Another nucleic acid fragment, aptamer is a single-stranded DNA or RNA that can bind with different targets, such as chemical molecules, RNA, DNA or protein with high affinity and specificity to block protein–protein or receptor–ligands interactions. Pegaptanib (macugen), The first PEGylated RNA aptamer drug, pegaptanib (macugen), was approved by FDA in 2004, binding to extracellular VEGF165 with high specificity and affinity [186]. It can be seen that these RNAs will also play a key role in the cancer treatment.

#### Delivery of therapeutic other nucleic acids

Exosomes can also deliver these nucleic acids for cancer therapy. For instance, Zheng et al. [187] transfected the lncRNA PTENP1 lentiviral vector into HEK293A cells, and then secreted exosomes contained PTENP1. Eventually the exosomal PTENP1 protected PTEN by sponging miR-17 and inhibited the biomalignant behavior of bladder cancer. Similarly, Zheng et al. [188] obtained exosomal circLPAR1 by the same methods, which could suppress colorectal cancer cell growth through suppressing BRD4 expression via METTL3-eIF3h interaction. In addition, aptamers are often used to modify exosomes to enhance their ability to target tumors. Exosomes derived from HEK293T cells were modified by A9g (PSMA) aptamer and loaded with survivin siRNA, which could be specifically delivered to tumors and effectively block tumor growth [143]. All in all, these system are emerging, and its their successful delivery will also contribute to cancer therapy.

## Exosome-based clinical applications for cancer treatment

As the discussed above, exosomes are a class of ideal drug delivery tool, which have also been performed in cancer clinical trials. The database www.ClinicalTrials.gov (accessed on April 2022), has been examined to assess the major exosomes’ clinical applications. 105 trials are registered within the study object “exosome” and “cancer”.

Table 4 summarizes the studies related to using “exosomes” for cancer therapy. Among them, immature DC-derived exosomes have been applied for melanoma and NSCLC with similar safety results. In addition, two clinical trials investigating plant-derived exosomes as cancer therapy are currently under way. At present, two clinical trials are on-going to study plant-derived exosomes for cancer treatment. In the first trial, grape-derived exosome-like nanoparticles are being tested for their effects on oral mucositis and related pain after radiotherapy and chemotherapy for head and neck cancer (NCT01668849). In the second study, plant-derived exosomes loaded with curcumin are being evaluated for their efficacy for treating colorectal cancer after oral administration (NCT01294072). The clinical studies on exosome-loaded nucleic acids for cancer treatment have been also “completed” or “ongoing”. For example, the phase I trial (NCT03608631) sponsored by the M.D. Anderson Cancer Center (Texas, USA) have investigated the use of MSC derived exosomes for the treatment of stage IV pancreatic cancer patients with KrasG12D mutation. The patients were injected with KrasG12D siRNA loaded into exosomes which targeted the oncogenic *KRAS* gene, reducing its expression in pancreatic tumors [154]. In addition, the clinical trials that tumor cell-derived exosome delivers ASO to treat malignant glioma of brain and neoplasms have been completed. In total, these exosome-based clinical applications for cancer treatment demonstrate the reliability of these delivery systems once again.

**Table 4:** Exosome-based clinical applications for cancer treatment from clinical trials.com and references

Cancer	Phase	Start year	Source of exosome	Therapeutic cargo	Status	Sponsor	Clinical trial number/Reference
Metastatic pancreas cancer with KrasG12D mutation	I	2018	Mesenchymal stromal cells	krasG12D siRNA	Ongoing	M.D. Anderson Cancer Center, Houston, Texas, United States	NCT03608631
Non-small cell lung cancer	II	2010	Dendritic cells	Metronomic cyclophosphamide	Completed	Institute Gustave Roussy, Villejuif, France	NCT01159288
Colon cancer	I	2011	Plant	Curcumin	Recruiting	University of Louisville Hospital, Louisville, Kentucky, United States	NCT01294072
Head and neck cancer	I	2012	Grape	Lortab, fentanyl patch, mouthwash	Active, not recruiting	James Graham Brown Cancer Center, Louisville, Kentucky, United States	NCT01668849
Malignant glioma of brain	I	2012	Tumor cells	IGF-1R antisense oligodeoxynucleotide	Completed	Thomas Jefferson University Hospital; Jefferson Hospital for Neurosciences, Philadelphia, Pennsylvania, United States	NCT01550523
Malignant glioma neoplasms	I	2015	Tumor cells	IGF-1R antisense oligodeoxynucleotide	Completed	Thomas Jefferson University Hospital, Philadelphia, Pennsylvania, United States	NCT02507583
Metastatic melanoma	I	2000	Autologous dendritic cell	Pulsed with MAGE 3 tumor peptides	Completed	Institute Curie, Paris, France	[189]
Non-small cell lung cancer	I	2000	Autologous dendritic cell	Pulsed with MAGE-A3, -A4, -A10, and MAGE-3DPO4 tumor peptides	Completed	Duke University Medical Center, Durham, NC, USA	[190]
Colorectal cancer	I	2006	Autologous ascites	The granulocyte–macrophage colony-stimulating factor	Completed	The Fourth Hospital Affiliated to Guangxi Medical University, Liuzhou, China	[191]

## Conclusions and future perspectives

Lower immunogenicity, lower toxicity, and better crossing biological barriers are the key advantages of exosomes over traditional nanocarriers. Based on these advantages, exosomes have shown great value in nucleic acid delivery, and can protect therapeutic substances from degradation and clearance by the host immune system. Additionally, the inherent targeting ability derived from their parental cells makes exosomes possess the potential of targeted delivery, enhancing the ability to penetrate the tumor vascular barrier and bioaccumulation at tumor sites, greatly improving their therapeutic efficacy. What’s more, therapeutic applications of exosomes as drug delivery vectors have been explored in numerous preclinical studies and several clinical trials. Thus, exosome-based delivery systems have unique advantages in cancer treatment. In this review, recent studies of using exosomes to deliver different nucleic acids (DNA, mRNA, miRNA, siRNA, circRNA, etc.) to treat various cancers are summarized.

Although significant progress has been made, some challenges hinder the exosomal therapeutic application. The first challenge is the large-scale production of exosomes for clinical trials. To increase the production of exosomes, bioreactors, 3D scaffolds, and microfluidic devices are adopted. For example, Haraszti et al. [192] applied 3D culture combined with tangential flow filtration (TFF) to increase the production of exosomes by 140-fold compared with 2D or 3D cultures or TFF. Another study found that using a hollow fiber bioreactor could increase the yield of exosomes by 40-fold [193]. Yang et al. [94] reported a cellular nanoporation (CNP) method to produce a large number of exosomes. The results revealed that compared with the traditional strategies (bulk electroporation and Lipo2000 transfection), CNP produced up to 50-fold more exosomes. The method of separation and purification of exosomes based on microfluidic devices also showed promising results [194–196]. Wang et al. [194] reported that a 3D nanostructured microfluidic chip could capture 90% of exosomes. In addition, some studies have shown that stress environments such as hypoxia, low pH and anticancer drugs could stimulate the production of more exosomes [197–199]. Also, food-derived exosomes, including bovine milk- and grape-derived exosomes, have shown promising results in preclinical studies [200, 201]. However, quality should be guaranteed while increasing output, especially considering the contamination or the size overlap between exosomes and other EVs.

The second challenge is to develop new methods for loading nucleic acids into exosomes to make up for defects of traditional methods. The low loading efficiency of current exosome-nucleic acid-loading strategies, including electroporation, incubation, and transfection, limits their application. For example, although electroporation is considered to be the best method for loading nucleic acids into exosomes, this method is easy to lead to the aggregation and degradation of nucleic acids and the change of exosome properties [202]. In addition, the simple incubation method is very limited in the type of the loaded cargo [203]. Transfection methods should further simplify the process and reduce the cost of mass production. To solve these problems, some potential new nucleic acid loading methods have been developed. For instance, Li et al. [91] constructed the fusion protein CD9-HuR through fusing the exosomal membrane protein CD9 with the RNA binding protein HuR, which would selectively enrich the target RNA into the exosomes. The results revealed that the specific RNA miRNA155 loaded into CD9-HuR modified exosomes was sevenfold higher than that of the control group. In addition, studies have shown that exosome-enriched RNAs shared three specific sequence motifs, including CAGUGAGC, UAAUCCCA, and ACCAGCCU, which played a role as cis-acting elements targeting to exosomes and helped us modify the exosomes to selectively enrich more candidate RNAs for therapeutic purposes [204]. Besides, it is necessary to develop new and efficient LNPs transfected with nucleic acid into exosomes. Wang et al. [205] developed a physical–chemical hybrid platform involving cationic LNPs exposed to cyclic stretch, which could effectively deliver siRNAs and plasmid DNAs. And the gene silencing efficiency was about 10% higher than that of commercial transfection reagents Lipo2000. Similarly, Hu et al. [206] developed thermostable ionizable lipid-like nanoparticles to deliver siRNA, which had good physical and chemical properties, thermal stability (not degraded at 40 °C for one week), and excellent siRNA transport efficiency (the same as that of Lipo2000). These techniques will provide new ideas for loading nucleic acid into exosomes.

The third challenge is to achieve personalized precision treatment of cancer. The heterogeneity of exosomes and complex in vivo environment limit the precise delivery and expected efficacy. To solve this problem, the cancer patient’s autologous exosomes can be the best choice of delivery carriers owing to the remarkable targeting ability against cancer cells. Among them, tumor cells can be obtained from minimally invasive and surgical samples and expanded in vitro under specific culture conditions. Gong et al. [207] domesticated tumor cells from gastric cancer patients under acidic conditions similar to the tumor tissue environment and found that the acidic environment would promote tumor cells to secrete more exosomes and improve the ability of these exosomes to be taken up by homologous gastric cancer cells. Futhermore, Wang et al. [208] obtained immune activated macrophage tumor hybrid cells by in vitro immune acclimation of patients’ own tumor cells and macrophages. The macrophage tumor chimeric exosomes secreted by these cells could inherit the functions of two kinds of source cells: (a) activating tumor specific immune response; (b) inheriting the homing ability of tumor cells and actively target tumor tissues. In addition, fluorescence, luminescence, PET-MRI and SPECT imaging techniques are used to track the biological distribution of exosomes and payloads, and obtain images with anatomical details, which will provide accurate and customized medical care for cancer patients [209].

Fourthly, considering that exosomes contain a group of discrete proteins and functional immune molecules, the application of exosomes may trigger a strong response of the host immune systems to a certain extent, resulting in the rapid disappearance of exosome-based drug delivery systems. Therefore, a comprehensive preclinical examination, including pharmacokinetics, toxicity characteristics and pharmacodynamics, should be performed to prevent potential side effects.

## Data Availability

Not applicable.

## Abbreviations

mRNA: Messenger RNA
miRNA: MicroRNA
siRNA: Small interfering RNA
circRNA: Circular RNA
EVs: Extracellular vesicles
LNPs: Lipid nanoparticles
SIRPα: Signal regulatory protein alpha
HEK293: Human embryonic kidney
DC: Dendritic cell
NSCLC: Non-small cell lung cancer
HEK: Human embryonic kidney
CaCl_2_: Calcium chloride
PC: Phosphatidylcholine
HMGN1: High mobility group nucleosome-binding protein 1
ASOs: Antisense oligonucleotides
FDA: Food and drug administration
exo-AAV: Exosome-associated adeno-associated virus
LHFPL5: lipoma HMGIC fusion partner-like 5
HA: hemagglutinin
PARP-1: poly (ADP-ribose) polymerase-1
MSC: Mesenchymal stem cell
EXOtic: EXOsomal transfer into cells
RBCs: Red blood cells
3′-UTR: 3′ untranslated region
AMOs: Anti-miRNA oligonucleotides
miR mimics: miRNA mimics
EMT: Epithelial–mesenchymal transition
OSCC: Oral squamous cell carcinoma
Dox: Doxorubicin
5-FU: 5-Fluorouracil
TAT: Transcriptional transactivator
dsRNA: Double stranded DNA
RISC: RNA-induced silencing complex
RNAi: RNA interference
RVG: Rabies virus glycoprotein
NK: Natural killer
BM-MSC: bone marrow mesenchymal stem cells (BM-MSC)
FAO: Fatty acid oxidation
CPT1A: Carnitine palmitoyltransferase 1A
TME: Tumor microenvironment
PDAC: Pancreatic ductal adenocarcinoma
OXA: Oxaliplatin
FGL1: Fibrinogen-like protein 1
TGF-β1: Transforming growth factor-β
EIciRNAs: Exon–intron circRNAs
snRNP: Small nuclear ribonucleoprotein
Pol II: Polymerase II
ORF: Open reading frame
EZH: Zeste homolog
lncRNA: Long noncoding RNA
shRNA: Short hairpin RNA
TFF: Tangential flow filtration
CNP: Cellular nanoporation
